# STAT3 and NF-κB are Simultaneously Suppressed in Dendritic Cells in Lung Cancer

**DOI:** 10.1038/srep45395

**Published:** 2017-03-28

**Authors:** Rui Li, Fang Fang, Ming Jiang, Chenguang Wang, Jiajia Ma, Wenyao Kang, Qiuyan Zhang, Yuhui Miao, Dong Wang, Yugang Guo, Linnan Zhang, Yang Guo, Hui Zhao, De Yang, Zhigang Tian, Weihua Xiao

**Affiliations:** 1 The CAS Key Laboratory of Innate Immunity and Chronic Disease, Innovation Center for Cell Signaling Network, School of Life Sciences, University of Science and Technology of China, Hefei, China; 2 Hefei National Laboratory for Physical Sciences at Microscale, Engineering Technology Research Center of Biotechnology Drugs, Anhui Province, University of Science and Technology of China, Hefei, China; 3 Department of Respiration, Second Affiliated Hospital of Anhui Medical University, Hefei, China; 4 Cancer and Inflammation Program, Center for Cancer Research, National Cancer Institute, Frederick National Laboratory for Cancer Research (FNLCR), Frederick, Maryland, USA

## Abstract

Tumour-induced dendritic cell (DC) dysfunction plays an important role in cancer immune escape. However, the underlying mechanisms are not yet fully understood, reflecting the lack of appropriate experimental models both *in vivo* and *in vitro*. In the present study, an *in vitro* study model for tumour-induced DC dysfunction was established by culturing DCs with pooled sera from multiple non-small cell lung cancer (NSCLC) patients. The results demonstrated that tumour-induced human monocyte-derived DCs exhibited systematic functional deficiencies. Transcriptomics analysis revealed that the expression of major functional cluster genes, including the MHC class II family, cytokines, chemokines, and co-stimulatory molecules, was significantly altered in tumour-induced DCs compared to that in control cells. Further examination confirmed that both NF-κB and STAT3 signalling pathways were simultaneously repressed by cancer sera, suggesting that the attenuated NF-κB and STAT3 signalling could be the leading cause of DC dysfunction in cancer. Furthermore, reversing the deactivated NF-κB and STAT3 signalling could be a strategy for cancer immunotherapy.

Lung cancer is one of the most common malignant tumours worldwide. More than 80% of lung cancer cases are non-small cell lung cancer (NSCLC). A high risk of metastasis in NSCLC indicates systemic anti-tumour immune deficiency^1^. An inhibitor of the immune checkpoint marker PD-1 showed a remarkably reduced risk of death compared to standard chemotherapy in NSCLC, demonstrating the importance of systematically disrupting the suppressive immune response^2^. The study of tumour infiltrating immune cells revealed that dendritic cells (DCs) infiltrating NSCLC were blocked at the immature stage, suggesting their ability to compromise tumour-specific immune responses^3^.

As specialized antigen-presenting cells (APCs), dendritic cells are crucial for the initiation of adaptive immune responses^4^,^5^. However, their antigen recognition, processing, and presenting functions are typically disrupted or blocked during cancer development^6^,^7^. Tumour-induced DC tolerance has been suggested as pivotal in immune evasion and cancer development^8^,^9^,^10^. Numerous studies have focused on tumour-induced DC dysfunction and the reversal of DC tolerance as potential biological adjuvants in cancer vaccines^11^,^12^,^13^. However, tumour-induced DCs exhibit thoroughly altered differentiation and function, and the reduction of DCs or their precursors makes it difficult to trace the abnormal alterations and molecular mechanisms involved^6^,^7^.

To date, several cytokines and growth factors involved in the abnormal differentiation and function of tumour-induced DCs, such as TGF-β, VEGF, and IL-10, have been identified^14^. TGF-β together with some chemokines can lead to the insufficient activation and improper polarization of DCs^15^. *In vivo* administration of VEGF in tumour-free mice can lead to impaired DC development^16^, and DCs from IL-10 transgenic mice suppress antigen presentation and IL-12 production^17^. However, reflecting the complexity of the tumour environment, only a number of tumour-derived factors interfere with DC function^18^. However, in many cases, the tumour environment is also associated with chronic inflammation, and several inflammation factors may also boost the differentiation and function of DCs^19^,^20^. These anti- and pro-DC activities eventually reach a dynamic balance in DC dysfunction^21^, and make it more complicated to identify the underlying mechanisms.

Furthermore, current experimental models of tumour-induced DC dysfunction remain imperfect. The most commonly used *in vivo* model involves tumour-infiltrating DCs (TIDCs) obtained from clinical samples or tumour-bearing mice^3^,^9^,^11^. Because of the low abundance of DCs in circulation and at the tumour site, along with individual variation, it is challenging to perform detailed analyses of the abnormal differentiation and function of TIDCs. Many *in vitro* models employ DCs generated from peripheral blood monocytes (MoDCs) or murine bone marrow progenitor cells (BMDCs), with tumour cell line conditional medium or specific factors added in cell culture, which may not well represent the complexity of the tumour environment. Therefore, building a proper experimental model of tumour-induced DC tolerance is urgently needed and may greatly accelerate mechanistic studies.

Here, by using lung cancer patients’ sera, we generated an *in vitro* model of tumour-induced DC dysfunction. In this model, the ability to initiate proper anti-tumour immune responses in DCs was systematically disrupted. Further transcriptomic analysis revealed that tumour-induced DCs harboured a unique gene profile. The disrupted upstream signalling in tumour cultured DCs, including the attenuated canonical NF-κB and STAT3 signalling pathways, may be the key reason. Taken together, these results indicate that the tumour environment manipulates DC functional deficiency by simultaneously attenuating canonical NF-κB and STAT3 signalling, leading to the abnormal transcription of downstream genes.

## Results

### Establishment of an *in vitro* model of tumour-induced DC deficiency

To establish an *in vitro* model of tumour-induced DC deficiency, we obtained the widely used MoDCs model, and sera from NSCLC patients were collected and pooled to represent the tumour environment. In this model, human monocytes separated from the peripheral blood of healthy donors were cultured with GM-CSF and IL-4 in the presence of sera from tumour patients or their healthy donor counterparts. Monocyte-derived dendritic cells (MoDCs) were subsequently collected 5–7 days later for further detection.

Considering that tumour sera might block the *in vitro* generation of MoDCs, the cells were dual labelled with lineage cocktail 1 (lin-1)/CD11c for population analysis. Lin-1 contained antibodies against CD3, CD14, CD16, CD19, CD20, and CD56 to distinguish DCs from lymphocytes, monocytes, eosinophils, and neutrophils^22^. FACS analysis revealed that over 97% of cells displayed a lin-1^−^ CD11c^+^ population, suggesting typical MoDC generation in both cancer and healthy groups (Supplementary Fig. S1). Furthermore, MoDCs cultured in the presence of tumour sera showed no increased cellular apoptosis compared to the healthy controls (Supplementary Fig. S1). These data indicated that the tumour sera culture did not affect the *in vitro* yield of MoDCs.

**Figure 1:**
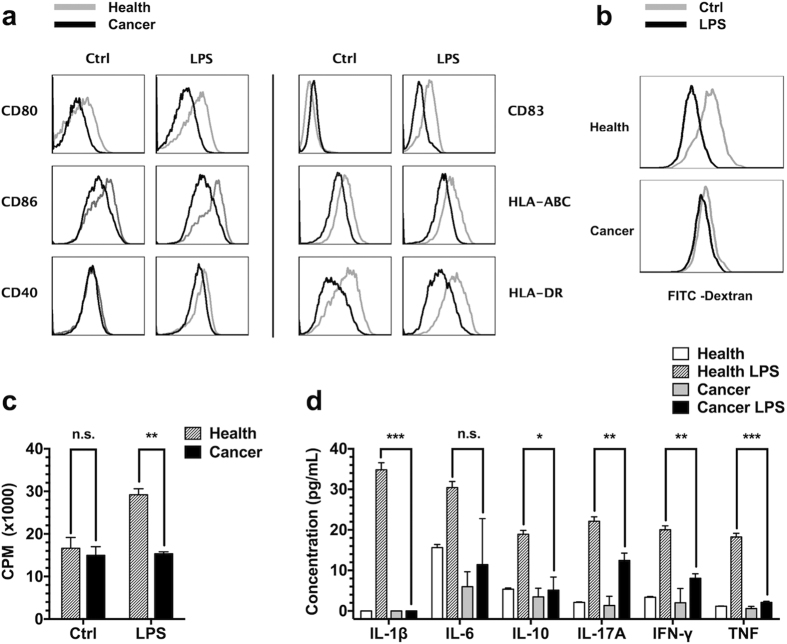
Tumour-induced DC deficiency *in vitro*. Human monocyte-derived DCs were generated and further incubated with or without LPS for an additional 2 days. (**a**) FACS assay for cell surface molecules of immature or mature DCs. Cancer serum cultured DCs are shown using a black line, and the healthy group is shown using a grey line. (**b**) DCs were incubated with FITC-labelled dextran. DC endocytosis was detected using flow cytometry and shown as indicated. (**c**) DCs induced lymphocyte proliferation. The DCs were co-cultured with PBMCs from another healthy donor at a ratio of 1:65. ^3^H-TdR was added on day 4, and beta liquid scintillation was measured 12–18 hours later. (**d**) DC cytokine secretion. Immature or mature DCs were further seeded into FCS medium without healthy or cancer sera, and the supernatant was collected and measured using BD CBA. Error bars, SEM. n.s., not significant; *P < 0.05; **P < 0.01; ***P < 0.001.

The phenotypes and functions of typical DCs were disrupted after tumour sera culture. Immature DCs cultured with tumour sera displayed decreased cell surface markers, such as co-stimulatory molecules and major histocompatibility complex (MHC) class I and II molecules. When the cells were further stimulated with lipopolysaccharide (LPS), the expression of these surface markers was slightly increased but remained lower than that observed in the healthy controls (Fig. 1a). Simultaneously, the endocytosis of immature DCs was down-regulated when cultured with tumour sera and changed little after LPS stimulation (Fig. 1b). In addition, a mixed lymphocyte reaction (MLR) showed that the DC-stimulated lymphocyte proliferation was significantly suppressed in the tumour sera group (Fig. 1c), likely reflecting reduced cell surface molecule expression (Fig. 1a) and cytokine secretion (Fig. 1d). In summary, MoDCs cultured with tumour sera exhibited systematic dysfunction, and antigen endocytosis, processing, presentation, and cytokine secretion were thoroughly disrupted. These data were consistent with the observed DC dysfunction *in vivo*^3^,^9^, suggesting that this *in vitro* model of tumour-induced DC deficiency well represented the *in vivo* observations.

### Overview of gene expression in tumour-induced DCs

**Figure 2:**
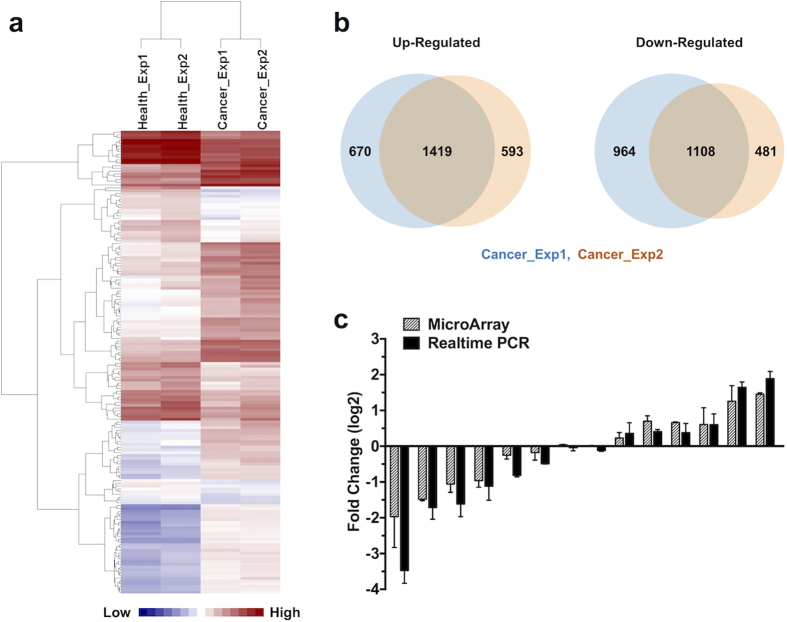
Overview of gene expression in tumour-induced DCs. (**a**) Heatmap of gene expression in DCs in the healthy controls or cancer group. Heatmap showing that DCs generated with different parallel tumour sera pools share similarities at the transcriptional level, while patterns differed between the cancer and healthy groups. (**b**) Venn diagram showing the overlap of differentially expressed genes within parallel cancer pools. (**c**) Diagram showing the fold-changes of both microarray and real-time PCR of several typical genes to confirm and validate the microarray data.

To reveal the mechanism involved in tumour-induced DC deficiency, immature DCs from cancer or healthy groups were collected, and mRNA expression profiling experiments were performed (GEO Series GSE84720). Initially, we explored the microarray results using hierarchical clustering. Heatmap analysis showed similar gene expression patterns within parallel tumour sera pools, while the patterns considerably differed between cancer and healthy groups (Fig. 2a). In addition, several mRNAs were selected, and real-time PCR was performed to confirm and validate the microarray data (Fig. 2c).

Gene expression profiles analysis found 1419 up-regulated and 1108 down-regulated differentially expressed genes (DEGs) in both parallel samples of tumour-induced immature DCs (Fig. 2b), and a list of the most significant DEGs is shown in Supplementary Table S1. The transcriptional level of HLA-DRA was markedly reduced, consistent with decreased cell surface MHC class II expression in tumour sera-cultured DCs (Fig. 1a). In addition, transcription of the chemokines CCL18 and CCL17 was decreased in the cancer group. Considering that these molecules attract different T-cell subtypes^23^,^24^, the reduced CCL18 and CCL17 may affect immune system activation and homeostasis at steady-state conditions.

### Major clusters of DEGs are altered in tumour-induced DCs

**Figure 3:**
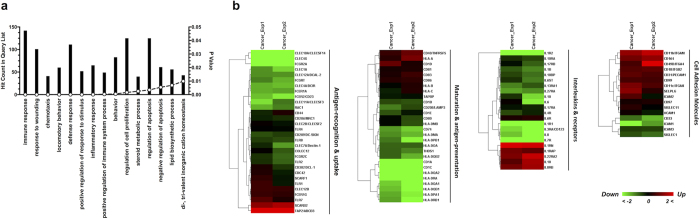
Clusters of DEGs altered in tumour-induced DCs. (**a**) Gene ontology enrichment analyses of significantly altered genes in tumour-induced DCs. The bar graph shows the numbers of genes and corresponding p-values of each category. (**b**) Heatmap for immune-related gene sets of DCs. Genes fold-changes were based on microarray data and shown in colour as indicated.

To assess the biological function of DEGs altered in tumour-induced DCs, gene ontology enrichment analyses were performed using DAVID bioinformatics resources^25^,^26^ (Fig. 3a). The most significant biological processes included immune response, response to wounding, and chemotaxis, indicating that various aspects of normal DC functions were affected in tumour-induced DCs. Moreover, KEGG pathway enrichment analyses of DEGs were performed based on DAVID tools, and the results are shown in Supplementary Table S2. The top KEGG pathways included haematopoietic cell lineage, antigen processing and presentation, and cell adhesion molecules (CAMs). Gene ontology and pathway enrichment analyses revealed systematic dysfunction in tumour-induced DCs, and these cells considerably differed from the normal function in initiating adaptive immune responses.

To further elucidate the crucial genes altered in tumour-induced DCs, the microarray data were analysed based on the immune-related gene sets obtained from the literature^27^ and online resources (QIAGEN resources, GO-Biological Process, etc.) (Fig. 3b and Supplementary Fig. S3). Most genes in the categories of antigen recognition, uptake, and presentation were down-regulated in tumour-cultured DCs. Particularly, the members of MHC class II families, Toll-like receptors (TLRs), and C-type lectin were decreased, indicating impaired antigen recognition and presenting functions. Moreover, the transcription of several interleukins and their receptors was suppressed, and many other cell surface CD antigens and adhesion molecules were up-regulated in tumour-induced DCs. The different patterns among the categories of immune-related genes provided a complete view of functional changes in tumour-induced DCs and demonstrated that antigen presentation as the major function of DCs was thoroughly suppressed, while communication with different cell subtypes diversely changed.

### Major clusters of signalling pathways are altered in tumour-induced DCs

**Figure 4:**
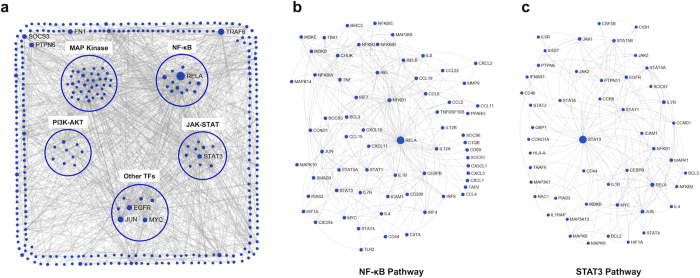
Altered signalling network of tumour-induced DCs. (**a**) Direct PPI network for immune-related DEGs and major signalling pathway genes through NetworkAnalyst analysis based on the InnateDB interactome PPI database. To obtain a detailed view of (A), sub-networks were constructed by selecting the nodes connected with RELA (**b**) or STAT3 (**c**).

To further reveal the key signalling pathways altered in tumour-induced DCs that lead to functional deficiency, immune-related DEGs and major signalling pathway genes were identified to construct a protein-protein interaction (PPI) network using NetworkAnalyst^28^,^29^ based on the InnateDB interactome PPI database^30^; the zero-order interaction network is shown in Fig. 4a. To obtain a general view of the grouped signalling pathways, NF-κB and STAT pathways were associated with inflammation, growth, and differentiation, and MAP kinase and PI3K-AKT pathways were associated with proliferation, survival, and motility. Other top transcription factors included EGFR, CEBPG, BCL2, JUN, MYC, and SPI1. Pathway enrichment showed alterations in multiple signalling pathways, implying diverse changes in the differentiation and function of DCs.

To identify high impact signalling pathways in the PPI network, the node degree and betweenness were sorted, and the top nodes are shown in Supplementary Table S3. Several members of the canonical NF-κB pathway were in the top list, including RELA, NFKB1, CHUK, IKBKB, IKBKG, and REL, indicating that the canonical NF-κB pathway was closely associated with immune-related DEGs in tumour serum-cultured DCs. Subsequently, a sub-network of RELA was constructed to obtain a detailed view, and the results are shown in Fig. 4b. Many differentially expressed cytokines, chemokines, receptors, and transcription factors were associated with the canonical NF-κB pathway, including IL-1β, IL-12A, IL-12B, TNF, CCL22, and CIITA. Among these genes, CIITA is the crucial trans-activator of MHC class II genes, and the down-regulation of CIITA may explain the impaired antigen presentation^31^. In addition to canonical NF-κB pathways, STAT1, STAT3, JUN, EGFR, and CEBPB were also on the top list, and these proteins may play crucial roles in tumour-induced DC signal transduction (Fig. 4c, Supplementary Fig. S4).

### NF-κB and STAT3 pathways are altered in tumour-induced DCs

**Figure 5:**
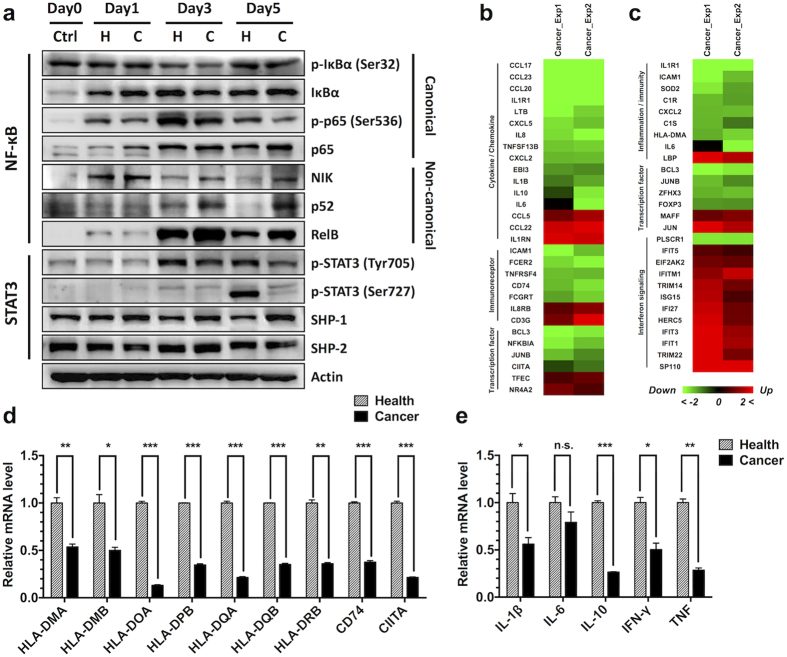
Altered NF-κB and STAT3 pathways in tumour-induced DCs. (**a**) Immunoblot analysis of NF-κB and STAT3 signalling during MoDC differentiation. The cells in the cancer or healthy control group were collected on days 1, 3, and 5 and subsequently detected through Western blotting with the indicated antibodies. Heatmap of the downstream immune-related target genes of canonical NF-κB (**b**) and STAT3 (**c**) signalling based on microarray data and shown in colour as indicated. Several targets genes, including antigen-presenting genes (**d**) and cytokines (**e**), were further validated using real-time PCR. Error bars, SEM. n.s., not significant; *P < 0.05; **P < 0.01; ***P < 0.001.

To validate the signalling pathway alterations in tumour-induced immature DCs, we selected NF-κB and STAT3 from the high impact signalling pathways for further verification. Down-stream target genes of the canonical NF-κB pathway were gathered from the literature^32^,^33^ and online resources (http://www.bu.edu/nf-kb/the-gilmore-lab/ and http://bioinfo.lifl.fr/NF-KB/), matched with DEGs in tumour-induced immature DCs, and categorized according to gene function (Fig. 5b). Most immune-related target genes of the canonical NF-κB pathway were down-regulated in tumour-induced DCs, including cytokines, chemokines, immunoreceptors, and several transcription factors, indicating that the canonical NF-κB pathway was attenuated in the cancer group. Moreover, many target genes of the STAT3 pathway^34^,^35^,^36^, categorized in inflammation, immunity, and transcription factors, were reduced in the cancer group (Fig. 5c). In addition, most genes in interferon signalling, which is negatively regulated through the STAT3 pathway, showed increased transcription levels in tumour-induced DCs (Fig. 5c). Taken together, these data suggested that STAT3 signalling is also blocked in the cancer group. To confirm the changes of downstream genes, we selected several targets genes vital to DC function, including antigen presentation genes and cytokines, and conducted verification using real-time PCR (Fig. 5d and Fig. 5e). The qRT results confirmed that these target genes were down-regulated at different levels in tumour-induced DCs. Downstream target gene analysis indicated that both canonical NF-κB and STAT3 pathways were attenuated in DCs cultured in tumour serum.

**Figure 6:**
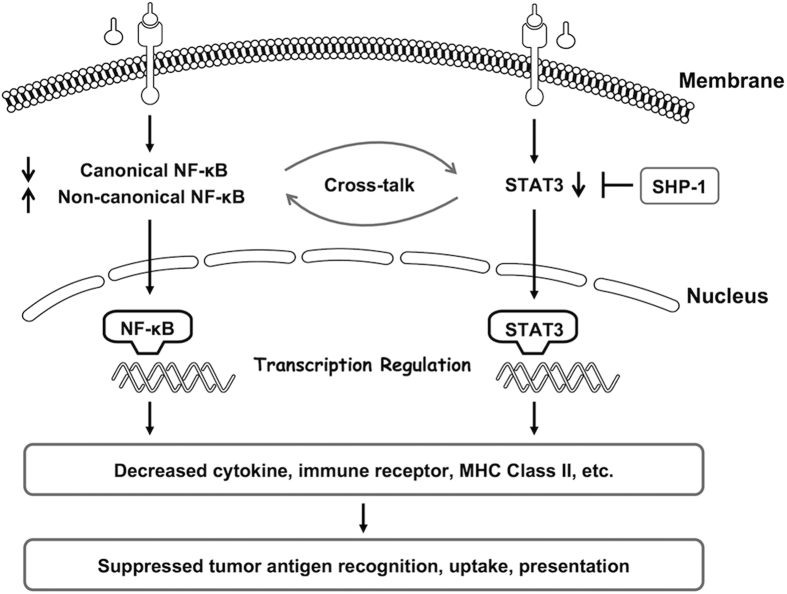
Schematic model of tumour-induced DC functional deficiency. MoDCs generated with tumour sera exhibited disrupted upstream signalling, including attenuated canonical NF-κB and STAT3 signalling, which might lead to the abnormal transcription of downstream genes, including decreased MHC class II family members, cytokine and chemokine profiles, and receptors, and further lead to impaired antigen recognition and presentation, undermining the initiation of proper anti-tumour immune responses.

To further validate alterations in NF-κB and STAT3 signalling pathways, the cells were harvested on days 1, 3, and 5 during MoDC differentiation. NF-κB and STAT3 signalling were analysed by immunoblotting (Fig. 5a). Canonical NF-κB signalling was continuously disrupted in tumour-induced DCs. The phosphorylation of both IκBα and p65 decreased from day 1 and was maintained at a low level during MoDC differentiation. However, the non-canonical NF-κB pathway showed increased signalling from day 3, and NIK, p52, and RelB were increased in the cancer group. STAT3 signalling analysis showed that the phosphorylation of STAT3 at Tyr705 was decreased in the cancer group from day 1, and STAT3 phosphorylation at Ser727 was also reduced, indicating that STAT3 signalling was blocked. SHP-1 is a SH2-containing tyrosine-specific protein phosphatase that binds STAT3 and mediates its dephosphorylation at Tyr705^35^,^37^. Western blotting showed that the expression of SHP-1 increased during DC differentiation, indicating that this protein may be involved in the inhibition of STAT3 signalling. In summary, immunoblot assay confirmed that both canonical NF-κB and STAT3 pathways were continuously attenuated in the cancer group during MoDC differentiation, which might lead to the abnormal transcription of downstream genes and the functional deficiency of DCs (Fig. 6).

## Discussion

In the present study, we established an *in vitro* model of tumour-induced DC dysfunction using sera obtained from NSCLC patients to represent the tumour environment. Malignant tumour patients, including NSCLC, exhibit systemic anti-tumour immune response deficiencies and show a high risk of tumour metastasis through blood circulation^19^. Typically, DCs and their precursors develop and are distributed within the circulation^38^,^39^,^40^, but these cells do not recognize tumour antigens and fail to initiate proper immune responses to eliminate the tumour cells. These observations strongly indicate that tumour-derived factors originating from the tumour site may also be distributed within the blood circulation and impair the differentiation and function of DCs^18^. According to this hypothesis, we employed the sera samples from NSCLC patients to simulate the effects of the tumour environment on the differentiation and function of DCs *in vitro*. To avoid different cancer stages or treatments that may interfere in this model, patients in late stage NSCLC were selected and sampled prior to tumour treatment. Subsequently, the collected sera were equally and randomly pooled into two groups to obtain a general view of their effects on DCs and to minimize individual variation.

After establishing the cell model, we performed multiple analyses to characterize these cells. In this model, cells cultured in the presence of tumour sera remained a typical lin-1^−^ CD11c^+^ MoDC population without increased apoptosis, indicating that the tumour sera culture did not directly affect the generation of MoDCs *in vitro*. However, tumour-induced DCs showed systematic functional deficiencies, including (I) decreased cell surface co-stimulatory molecules and MHC class I and II molecules, (II) impaired endocytosis by immature DCs, (III) reduced cytokine secretion from mature DCs, and (IV) suppressed DC-stimulated lymphocyte proliferation. In conclusion, MoDCs cultured with tumour sera lost their major functions as antigen-presenting cells, thereby preventing the initiation of proper immune responses against tumour cells. Moreover, we observed morphological changes in the cells in the tumour group, including cell elongation and decreased cellular aggregation (Supplementary Fig. S2). Subsequently, we performed FACS detection of CAMs, and the results showed that cancer group cells had increased CD18 (ITGB2), CD106 (VCAM-1), and CD31 (PECAM-1), with decreased CD54 (ICAM-1) (Supplementary Fig. S2). The altered CAM pattern (Fig. 3b and Supplementary Fig. S2) might explain the morphological changes of DCs *in vitro* and may also affect cellular communication between DCs and other cells.

During the microarray analysis process, the most remarkable alteration in tumour-induced DCs was the decrease of antigen-presenting genes, and nearly all MHC class II genes showed significant decreases in the cancer group, including the classical MHC class II genes (HLA-DP, HLA-DR, and HLA-DQ), HLA-DM, and HLA-DO (Fig. 5d). Moreover, the CD74 gene, encoding the molecular chaperone invariant chain, was also decreased in the cancer group. The transactivator protein CIITA plays an essential role in the transcriptional regulation of MHC class II genes and non-MHC genes, including CD74^41^,^42^, and its significant reduction in tumour-induced DCs (Fig. 5d) might explain the decreased expression of antigen-presenting genes. We further analysed the transcriptional regulation of CIITA, which likely occurs through activated transcriptional complexes, including PU.1, SP1, IRF8, and NF-κB^31^. In the present study, canonical NF-κB signalling was continuously attenuated in the cancer group during MoDC differentiation (Fig. 5a), likely resulting in the transcriptional suppression of CIITA and MHC class II genes and the further impairment of antigen presentation (Supplementary Fig. S5).

Signalling pathway enrichment analysis of tumour-induced DCs revealed that multiple signalling pathways associated with inflammation, growth, differentiation, proliferation, survival, and motility were diversely changed. We selected NF-κB and STAT3 from the high impact signalling pathways to perform further verification. The canonical NF-κB and STAT3 signalling in tumour-induced DCs was continuously disrupted and maintained at a low level during MoDC differentiation, while non-canonical NF-κB signalling was activated from day 3. These signalling alterations may be crucial for the transcriptional suppression of vital cytokines and antigen-presenting genes, leading to the functional deficiency of DCs. However, this signalling dysregulation was only partially described, reflecting the complicated mechanism involved, such as the disruption of upstream signalling, ubiquitin-mediated degradation^43^, noncoding RNA-mediated regulation^44^, and crosstalk among these factors. Generally, NF-κB and STAT3 pathways can separately or together regulate various downstream target genes, including cytokines, chemokines, receptors, and transcription factors, and some of the downstream targets may in turn further trigger the signalling of either pathway^45^,^46^,^47^. Furthermore, these pathways occasionally undergo antagonistic interactions, forming a negative regulator. The closely intertwined crosstalk makes it challenging to determine the underlying mechanism and to manipulate signals that either block or boost signalling. As for the attenuated canonical NF-κB and STAT3 signalling in tumour-induced DCs in the model used in the present study, these two pathways may cooperate to promote DC development. Insights into the molecular mechanisms may help us understand and reverse tumour-induced DC dysfunction.

The precise tumour sera-derived factors that induced DC dysfunction remain unknown. We detected several preliminary cytokines and growth factors using BD CBA technology and observed that both pro-inflammatory and anti-inflammatory cytokines, including IL-1β, IL-2, IL-4, IL-6, IL-8, IL-10, IL-17A, TNF, VEGF, and TGF-β, were expressed at higher levels in tumour sera. This finding is consistent with the understanding that the tumour environment is associated with chronic inflammation, and anti- and pro- inflammatory activities might eventually lead to DC dysfunction. Moreover, tumour sera also contained several chemokines and soluble ligands, such as sICAM-1 and sVCAM-1, which might also play roles in tumour-induced DC functional deficiency. Using the new techniques and methods in proteomics, more components might be identified in the tumour environment to obtain an understanding of their roles in tumour immune evasion^18^.

The other question that needs further discussion is how to define or describe tumour-induced DCs using the model in the present study. Although these cells remained a typical induced MoDC population, their antigen-presenting function was thoroughly disrupted. Moreover, the gene expression pattern of interleukins, chemokines, cell surface CD antigens, CAMs, ligands, and receptors were diversely altered. Whether these cells remained anergic or acquired tolerogenic and/or immunosuppressive activities^48^,^49^,^50^ is unknown. The preliminary analysis of the surface inhibitory receptors of DCs showed that several receptors, including B7H3, Tim3, CD31, and OX2, were increased in tumour-induced DCs (Supplementary Table S4), with a decrease in CD301, FcγRIIA, DCIR, ILT4, and Tyro3. Other inhibitory receptors on DCs, such as B7H1, B7H4, and ILT3, showed no significant changes. Further screening and verification of the inhibitory receptors will expand the current understanding of tumour-induced DC dysfunction. More importantly, the targeted blockage of inhibitory receptors will help to manipulate DC function and serve as a cancer vaccine in immunotherapy^51^,^52^,^53^.

In summary, we constructed an *in vitro* model of tumour-induced DC dysfunction using sera from NSCLC patients to represent the tumour environment. In this model, systematic functional deficiency of DCs was achieved, leading to failures in tumour antigen recognition and the initiation of proper immune responses against tumour cells. High-throughput microarray analysis revealed that tumour-induced DCs had abnormal functional gene expression, including cytokines, chemokines, immune receptors, and MHC class II genes. Further upstream analysis revealed that cell signalling was disrupted in DCs from the cancer group, including attenuated canonical NF-κB and STAT3 signalling, which might result in abnormal target gene transcription (Fig. 6). Insights into the molecular mechanisms of tumour-induced DCs may provide an understanding of DC-dependent tumour immune escape, thereby enabling the manipulation of inhibitory signals and the promotion of cancer immune surveillance.

## Materials and Methods

### Patients and samples

The present study was submitted and approved through the Ethics Committee of the University of Science and Technology of China (No. USTCEC200800003) and conducted in accordance with the Declaration of Helsinki. Informed consent was obtained from all participants according to the guidelines established by the Institutional Review Board of Affiliated Hospital of Anhui Medical University. Patients in late stage NSCLC were selected and sampled prior to tumour treatment. There were a total of 56 patients, including 41 males and 15 females, aged 44–76 years. The patient sera were equally and randomly pooled into two groups to obtain a general view of its effects on DCs and to minimize individual variation. In the control group, blood plasma samples were collected from healthy donors at the Blood Centre of Anhui Province (Hefei, China). All blood serum or plasma samples were incubated at 56 °C in a water bath for 30 min to deactivate complement activity prior to use in cell growth medium.

### Monocyte isolation and DC culture

Human peripheral blood mononuclear cell (PBMC) were isolated from the peripheral blood of healthy donors at the Blood Centre of Anhui Province using Ficoll-Hypaque density gradient centrifugation (Solarbio, Beijing, China). Subsequent Percoll density gradient centrifugation (GE Healthcare, Chicago, IL, USA) was performed for monocyte separation as previously reported^54^. Human monocyte-derived DCs were generated after culturing the monocytes with rhGM-CSF (50 ng/mL, PeproTech, Rocky Hill, NJ, USA) and rhIL-4 (50 ng/mL, PeproTech) in RPMI 1640 medium (RPMI 1640 with 2 mM glutamine, 25 mM HEPES, 100 U/ml penicillin, and 100 mg/ml streptomycin) with the addition of sera from cancer patients or healthy controls, followed by incubation at 37 °C in 5% CO_2_ for 5 days. To obtain mature DCs, the cells were cultured in the same cytokine mixtures in the presence of LPS (50 ng/mL, Sigma-Aldrich, St. Louis, MO, USA) for an additional 2 days.

### Flow cytometry

For cell surface molecular detection, the cultured cells were harvested and coated with antibodies (FITC-anti-CD86, PE-anti-CD83, FITC-anti-HLA-DR, FITC-Lineage Cocktail 1 (lin-1), PE-anti-CD11c, Alexa-488-anti-CD31, FITC-anti-CD18, PE-anti-CD54, and FITC-anti-CD106 (BD Biosciences, San Jose, CA, USA), PerCP-Cy5.5-anti-CD40, PE-anti-CD80, and APC-anti-HLA-ABC (BioLegend, San Diego, CA, USA), or their isotypes (BD Biosciences and BioLegend) and measured through flow cytometry using a BD FACSCalibur.

For cell endocytosis detection, the cells were collected, washed, and resuspended at 1 × 10^5^ cells/mL in RPMI 1640 medium containing 10% FBS. Then, 500 μg/mL FITC-labelled dextran (Sigma-Aldrich) was added, followed by incubation at 37 °C (or 4 °C for the control group) in 5% CO_2_ for 1 hour. The cells were washed with cold PBS and subsequently detected using a BD FACSCalibur.

For cell apoptosis detection, the cells were harvested and resuspended in apoptosis binding buffer, followed by incubation with FITC-labelled Annexin V (BioLegend) and PI (Beckman Coulter, Brea, CA, USA) and subsequent detection using a BD FACSCalibur.

For cell cytokine detection, the cells were collected, washed, and seeded at 3 × 10^3^ cells/well onto 96-well plates in RPMI 1640 medium containing rhGM-CSF, rhIL-4, and 10% FBS for an additional 5 days to collect the supernatant. The cytokines were further measured using a BD CBA kit (Human Inflammatory Cytokines Kit and Human Th1/Th2/Th17 Cytokine Kit) and a BD FACSCalibur.

### Allogeneic mixed lymphocyte reaction

To detect the function of DCs in stimulating allogeneic lymphocyte proliferation *in vitro*, human monocyte-derived DCs were co-cultured with PBMCs obtained from another healthy donor at a 1:65 ratio in 96-well plates in RPMI 1640 medium containing rhGM-CSF, rhIL-4, and 10% FBS for an additional 5 days. Tritiated thymidine (^3^H-TdR, PerkinElmer, Waltham, MA, USA) was added at 1 μCi/well on day 4, and the cells were collected and measured using a TriCarb Liquid Scintillation Analyser (PerkinElmer) 12–18 hours later.

In the MHC Class II antibody competitive blocking experiment, 5 μL/mL of purified mouse anti-human HLA-DR (BD Biosciences) or mouse IgG2a, κ isotype control (BD Biosciences) was added to the DC-PBMC co-culture system, and 1 μCi/well ^3^H-TdR was added on day 4 and measured using a TriCarb Liquid Scintillation Analyser (PerkinElmer) 12–18 hours later.

### Gene expression profiling

Immature DCs cultured with tumour sera or healthy controls were collected, washed and stored in TRIzol reagent (Invitrogen, Carlsbad, CA, USA) at −80 °C, and gene expression profiling was performed at Chipscreen Biosciences (Shenzhen, China). Total RNA was isolated, and T7 oligo(dT) primer and reverse transcriptase were used to synthesize first-strand cDNA. The second cDNA strand was subsequently synthesized by using the first cDNA strand together with RNase H and DNA polymerase. The double-stranded cDNA was purified and used as the template for *in vitro* transcription in the presence of T7-RNA polymerase. The aRNA was subsequently purified and labelled with CY5 (healthy group) or CY3 (cancer group) followed by further purification, and gene expression profiling was performed based on the Operon Human Genome Array-Ready Oligo Set^TM^ (Version 4.0).

For the microarray analysis, 22,484 unigene data were selected, and the fold-changes were calculated using BRB-ArrayTools (http://brb.nci.nih.gov/BRB-ArrayTools.html) with LOWESS normalization. Next, DAVID Bioinformatics Resources^25^,^26^ (The Database for Annotation, Visualization and Integrated Discovery, http://david.abcc.ncifcrf.gov) were used to perform Gene Ontology (GO)-based functional enrichment and KEGG pathway enrichment analyses. For immune-related gene analysis, hierarchical clustering was performed by Cluster 3.0 based on complete linkage and visualized with TreeView software. Furthermore, a protein-protein interaction (PPI) network was constructed using NetworkAnalyst^28^,^29^ (http://www.networkanalyst.ca/NetworkAnalyst/faces/home.xhtml) based on the InnateDB interactome PPI database^30^.

### Quantitative Real-Time PCR

Total RNA was isolated using TRIzol reagent (Invitrogen), and cDNA was synthesized using M-MLV Reverse Transcriptase (Invitrogen) according to the manufacturer’s instructions. Quantitative real-Time PCR was performed on a StepOne PCR System (Applied Biosystems, Foster City, CA, USA) with FastStart Universal SYBR Green Master (Roche, Basel, Switzerland) according to the manufacturer’s instructions, and the primer pairs (Sangon, Shanghai, China) are listed in Supplementary Table S5.

### Immunoblot analysis

The cells were harvested, washed, and lysed with RIPA lysis buffer containing a protease inhibitor cocktail (BBI Life Sciences, Shanghai, China) together with NaF and Na_4_P_2_O_7_, and the protein concentration was measured using a BCA Protein Assay Kit (Pierce, Waltham, MA, USA). Subsequently, the samples were loaded onto SDS-PAGE gels and separated through electrophoresis. The proteins were transferred onto PVDF membranes and incubated with the corresponding primary Abs and HRP-conjugated secondary Abs. Chemiluminescent detection was performed using Alliance 4.7 (UVITEC Cambridge, Cambridge, UK) with Luminata Forte Western HRP Substrate (Millipore, Billerica, MA, USA).

The following antibodies were used: Phospho-IκBα (Ser32) (CST#2859, Cell Signaling Technology, Danvers, MA, USA), IκBα (CST#4812), Phospho-NF-κB p65 (Ser536) (CST#3033), NF-κB p65 (CST#3034), NIK (CST#4994), NF-κB2 p100/p52 (CST#4882), RelB (CST#4922), Phospho-Stat3 (Tyr705) (CST#9131), Phospho-Stat3 (Ser727) (CST#9134), SHP-1 (CST#3759), SHP-2 (CST#3397), Stat-3 (ab68153, Abcam, Cambridge, UK), β-Actin (ACTB) (BM0627, Boster, Wuhan, China), HRP Anti-rabbit IgG (CST#7074), and HRP anti-mouse IgG (BioLegend, 405306).

### Statistical analysis

A t-test was used to statistically compare the differences between the two groups using Prism software (GraphPad Software, Inc., La Jolla, CA, USA). The data are shown as the mean ± SEM. A p-value less than 0.05 was considered to be the threshold for statistical significance, and the p-value is indicated as either n.s., not significant; *P < 0.05; **P < 0.01; or ***P < 0.001.

## Additional Information

**How to cite this article**: Li, R. *et al*. STAT3 and NF-κB are Simultaneously Suppressed in Dendritic Cells in Lung Cancer. *Sci. Rep.* **7**, 45395; doi: 10.1038/srep45395 (2017).

**Publisher's note:** Springer Nature remains neutral with regard to jurisdictional claims in published maps and institutional affiliations.

## Supplementary Material

Supplementary Information
