# Supplementary material for: STAT3 and NF-κB are Simultaneously Suppressed in Dendritic Cells in Lung Cancer
Source: Sci Rep. 2017 Mar 28;7:45395. doi: 10.1038/srep45395 (PMC5368983; doi:10.1038/srep45395)
Supplement: Supplementary Information [file srep45395-s1.pdf]

# Supplementary Information

**STAT3 and NF-κB are Simultaneously Suppressed in Dendritic Cells in Lung Cancer**

Rui Li[1, 2], Fang Fang[1, 2, *], Ming Jiang[1, 2], Chenguang Wang[1, 2], Jiajia Ma[1, 2], Wenyao Kang[1, 2], Qiuyan Zhang[1, 2], Yuhui Miao[1, 2], Dong Wang[1, 2], Yugang Guo[1, 2], Linnan Zhang[1, 2], Yang Guo[1, 2], Hui Zhao[3], De Yang[4], Zhigang Tian[1, 2], and Weihua Xiao[1, 2, *]

[1] *The CAS Key Laboratory of Innate Immunity and Chronic Disease, Innovation Center for Cell Signaling Network, School of Life Sciences, University of Science and Technology of China, Hefei, China*

[2] *Hefei National Laboratory for Physical Sciences at Microscale, Engineering Technology Research Center of Biotechnology Drugs, Anhui Province, University of Science and Technology of China, Hefei, China*

[3] *Department of Respiration, Second Affiliated Hospital of Anhui Medical University, Hefei, China*

[4] *Cancer and Inflammation Program, Center for Cancer Research, National Cancer Institute, Frederick National Laboratory for Cancer Research (FNLCR), Frederick, Maryland, USA*

*Correspondence should be addressed to

W. X., 443 Huangshan Road, School of Life Sciences, University of Science and Technology of China, Hefei, China, 20027. Email: xiaow@ustc.edu.cn. Tel: +86 551 6360 6294. Fax: +86 551 6360 0535.

OR F.F., 443 Huangshan Road, School of Life Sciences, University of Science and Technology of China, Hefei, China, 20027. Email: fangfang@mail.ustc.edu.cn. Tel: +86 551 6360 0536. Fax: +86 551 6360 0535.

# Supplementary Figures & Tables

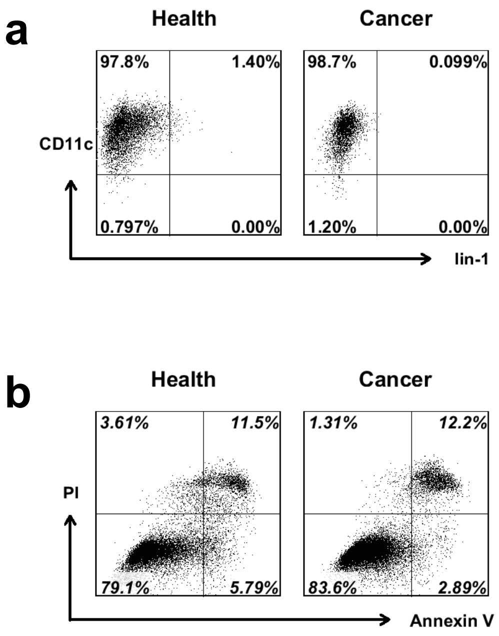


**Figure S1. Tumour-induced DCs maintained normal differentiation.** Human MoDCs were generated in health or cancer group. (a) FACS assay for DC population analysis. Cells were dual labeled with lin-1/CD11c, and FACS data showed typical MoDC generation in both cancer and healthy groups. (b) Apoptosis assay for DCs. Cells were harvested and incubated with AnnexinV-FITC and PI. Dot Plot showed MoDCs cultured in the presence of tumour sera showed no increased apoptosis compared to the healthy controls.

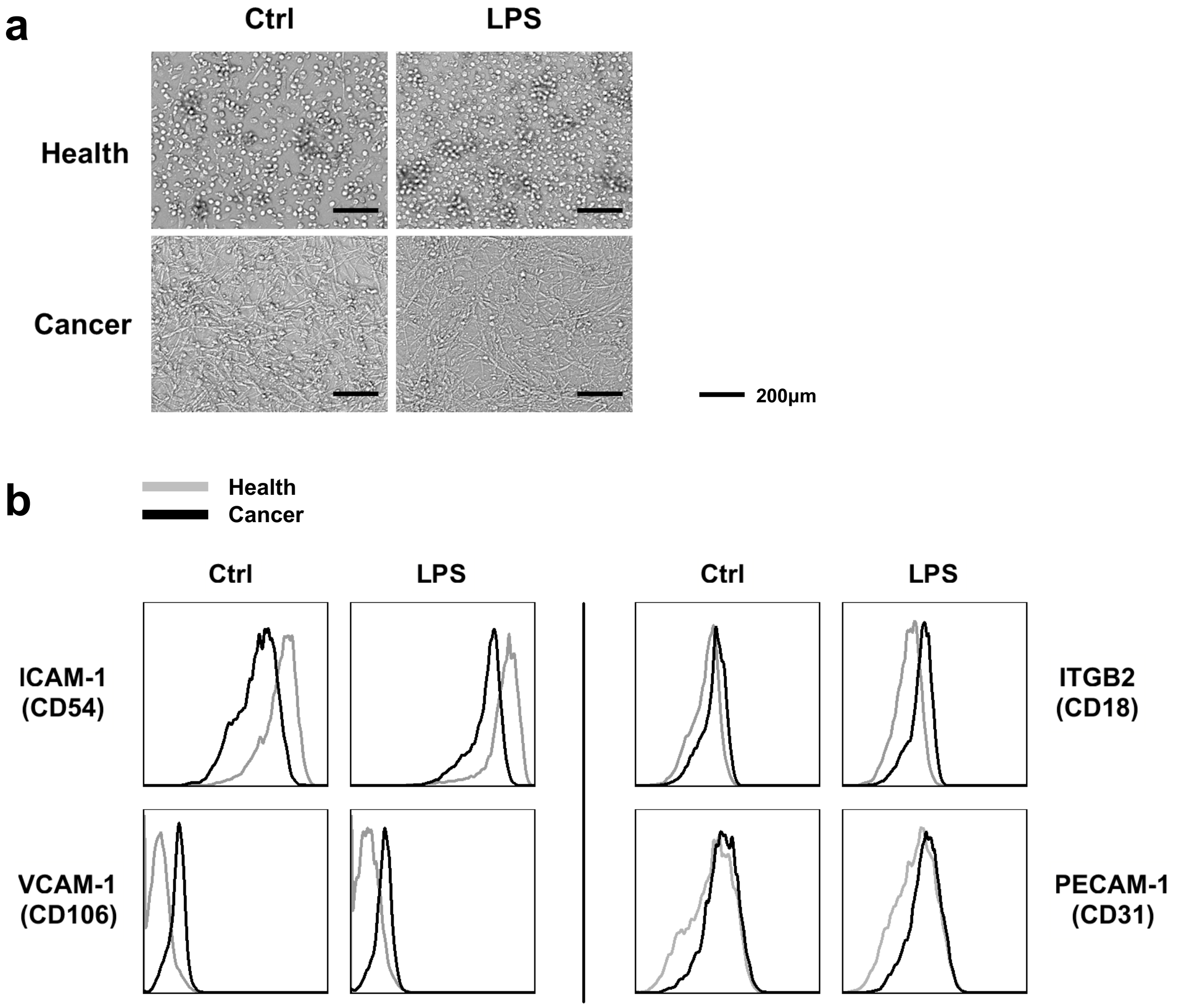


**Figure S2. Tumour-induced DCs morphology changes.** Human MoDCs were generated and further incubated with or without LPS for an additional 2 days. (a) Morphology of DCs in cancer or healthy group. Scale bars represent 200μm. (b) FACS assay for cell surface molecules of immature or mature DCs as indicated.

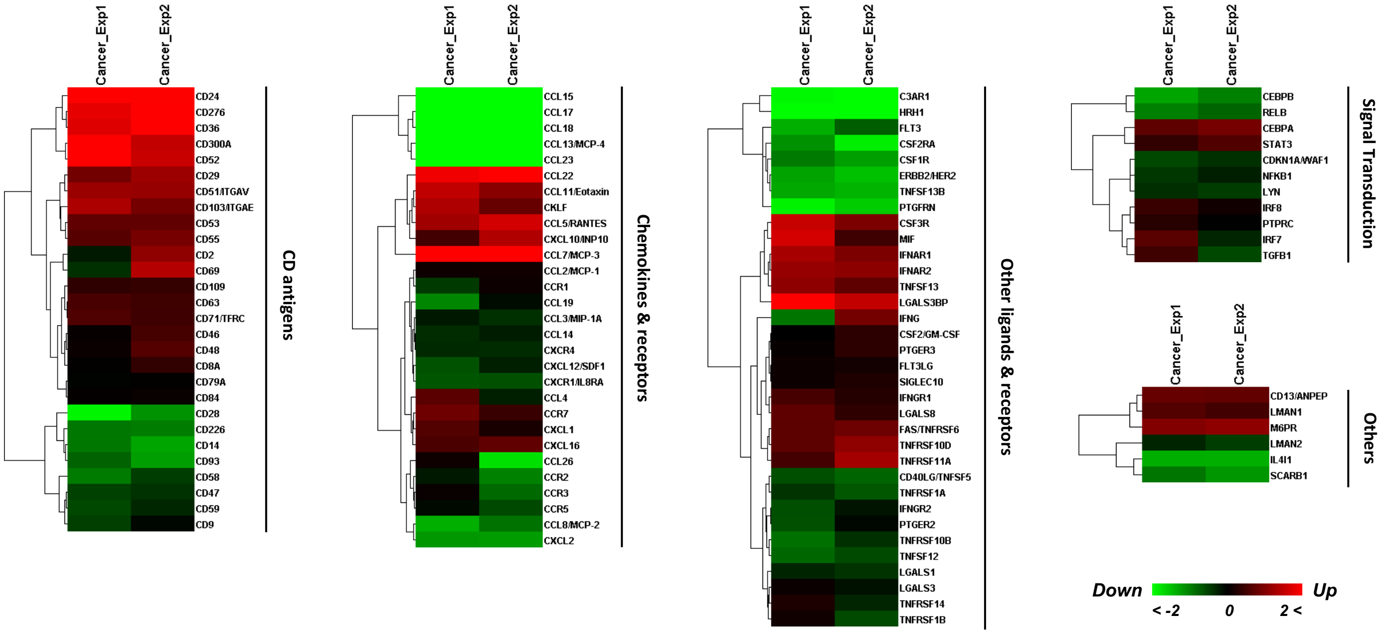


**Figure S3. Clusters of immune-related DEGs altered in tumour-induced DCs.** Heatmap for immune-related gene sets of DCs. Genes fold-changes were based on microarray data and shown in colour as indicated.

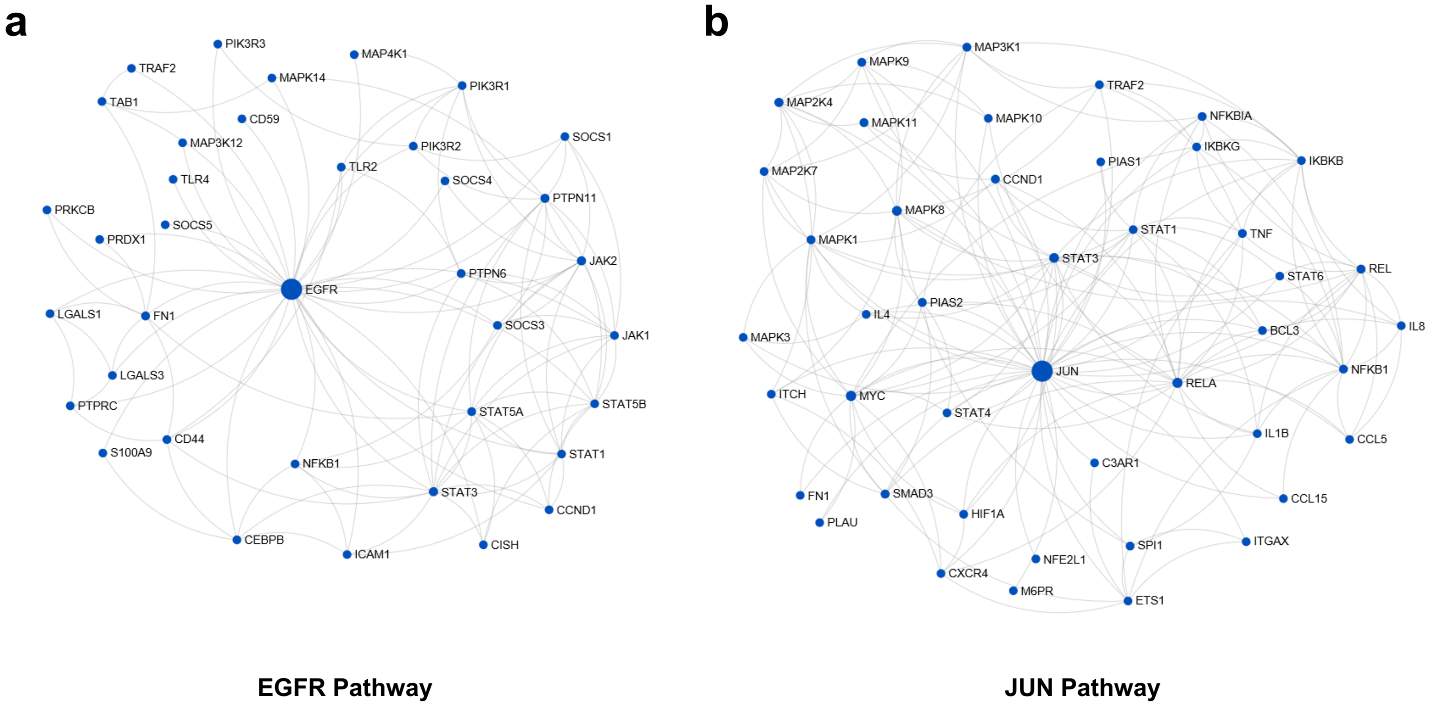


**Figure S4. Sub-network of EGFR & JUN signaling pathways.** To obtain a detailed view of Figure 4a, sub-networks were constructed by selecting the nodes connected with EGFR (a) or JUN (b).

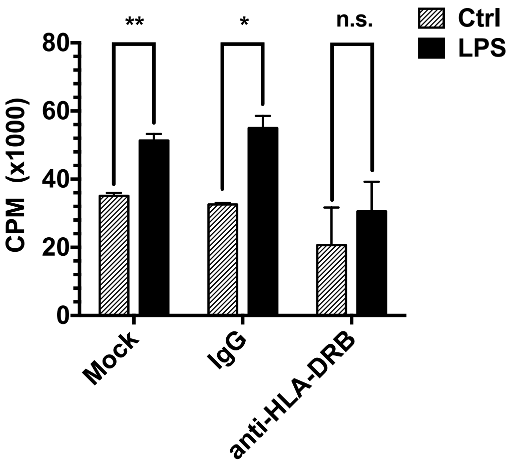


**Figure S5. Competition MLR by HLA-DR blockade.** MoDCs generated in FCS medium were co-cultured with PBMCs from another healthy donor at a ratio of 1:65, with HLA-DR blockading antibody or isotype IgG added. $^{3}$H-TdR was added on day 4, and beta liquid scintillation was measured 12-18 hours later. Error bars, SEM. n.s., none significant; *, P < 0.05; **, P < 0.01.

**Table.S1. List of genes found to be significantly regulated in microarray experiments**

| *Gene ID* | *Symbol* | *Location* | *Full Name* | *Cancer Exp1* | *Cancer Exp2* |
|---|---|---|---|---|---|
| *Top 20 down-regulated genes* | | | | | |
| 6414 | SEPP1 | 5q31 | Selenoprotein P, Plasma, 1 | 0.0329 | 0.0567 |
| 7850 | IL1R2 | 2q12-q22 | Interleukin 1 Receptor, Type II | 0.0536 | 0.0243 |
| 339390 | CLEC4G | 19p13.2 | C-Type Lectin Domain Family 4, Member G | 0.0993 | 0.0919 |
| 6362 | CCL18 | 17q11.2 | Chemokine (C-C Motif) Ligand 18 (Pulmonary And Activation-Regulated) | 0.1227 | 0.2583 |
| 283316 | CD163L1 | 12p13.3 | CD163 Molecule-Like 1 | 0.1339 | 0.0752 |
| 5031 | P2RY6 | 11q13.5 | Pyrimidinergic Receptor P2Y, G-Protein Coupled, 6 | 0.1341 | 0.1066 |
| 6361 | CCL17 | 16q13 | Chemokine (C-C Motif) Ligand 17 | 0.1549 | 0.2886 |
| 23179 | RGL1 | 1q25.3 | Ral Guanine Nucleotide Dissociation Stimulator-Like 1 | 0.1572 | 0.1730 |
| 10462 | CLEC10A | 17p13.1 | C-Type Lectin Domain Family 10, Member A | 0.1577 | 0.1228 |
| 3957 | LGALS2 | 22q12-q13\|22q13.1 | Lectin, Galactoside-Binding, Soluble, 2 | 0.1588 | 0.1544 |
| 27076 | LYPD3 | 19q13.31 | LY6/PLAUR Domain Containing 3 | 0.1604 | 0.3603 |
| 6035 | RNASE1 | 14q11.2 | Ribonuclease, RNase A Family, 1 (Pancreatic) | 0.1712 | 0.1557 |
| 3269 | HRH1 | 3p25 | Histamine Receptor H1 | 0.1841 | 0.1603 |
| 3122 | HLA-DRA | 6p21.3 | Major Histocompatibility Complex, Class II, DR Alpha | 0.1954 | 0.1637 |
| 50486 | G0S2 | 1q32.2-q41 | G0/G1 Switch 2 | 0.2000 | 0.2014 |
| 4318 | MMP9 | 20q11.2-q13.1 | Matrix Metallopeptidase 9 | 0.2006 | 0.2749 |
| 64240 | ABCG5 | 2p21 | ATP-Binding Cassette, Sub-Family G (WHITE), Member 5 | 0.2059 | 0.1597 |
| 85019 | TMEM241 | 18q11.2 | Transmembrane Protein 241 | 0.2092 | 0.1686 |
| 1471 | CST3 | 20p11.21 | Cystatin C | 0.2156 | 0.1570 |
| 2162 | F13A1 | 6p25.3-p24.3 | Coagulation Factor XIII, A1 Polypeptide | 0.2192 | 0.1939 |
| *Top 20 up-regulated genes* | | | | | |
| 440992 | RPS20P14 | 3q27.3 | Ribosomal Protein S20 Pseudogene 14 | 13.6692 | 9.5598 |
| 118430 | MUCL1 | 12q | Mucin-Like 1 | 12.8619 | 12.2210 |
| 390107 | RPS24P15 | 11p13 | Ribosomal Protein S24 Pseudogene 15 | 9.6442 | 6.2639 |
| 130773 | RPL23AP37 | 2p14 | Ribosomal Protein L23a Pseudogene 37 | 9.3945 | 5.4780 |
| 6286 | S100P | 4p16 | S100 Calcium Binding Protein P | 7.9978 | 6.0193 |
| 348 | APOE | 19q13.2 | Apolipoprotein E | 7.9822 | 8.9974 |
| 54627 | MAP10 | 1q42.2 | Microtubule-Associated Protein 10 | 7.5633 | 3.7377 |
| 55711 | FAR2 | 12p11.22 | Fatty Acyl CoA Reductase 2 | 7.2273 | 7.8296 |
| 27306 | HPGDS | 4q22.3 | Hematopoietic Prostaglandin D Synthase | 7.2152 | 7.5915 |
| 441533 | RPL26P37 | Yp11.2 | Ribosomal Protein L26 Pseudogene 37 | 7.0451 | 3.9103 |
| 4828 | NMB | 15q22-qter | Neuromedin B | 6.6957 | 6.0493 |
| 10114 | HIPK3 | 11p13 | Homeodomain Interacting Protein Kinase 3 | 6.6845 | 4.0924 |
| 341356 | RPL31P50 | 12p11.21 | Ribosomal Protein L31 Pseudogene 50 | 6.6412 | 2.9488 |
| 253013 | RPL31P8 | 5q12.1 | Ribosomal Protein L31 Pseudogene 8 | 6.5690 | 3.7312 |
| 597 | BCL2A1 | 15q24.3 | BCL2-Related Protein A1 | 6.4727 | 3.9652 |
| 314 | AOC2 | 17q21 | Amine Oxidase, Copper Containing 2 (Retina-Specific) | 6.4507 | 8.3630 |
| 432369 | ATP5EP2 | 13q12 | .TP Synthase, H+ Transporting, Mitochondrial F1 Complex, Epsilon Subunit Pseudogene | 6.3876 | 3.2278 |
| 112942 | CFAP36 | 2p16.1 | Cilia And Flagella Associated Protein 36 | 6.2681 | 4.8231 |
| 126235 | RPS4XP21 | 19q13.11 | Ribosomal Protein S4X Pseudogene 21 | 6.2045 | 3.9817 |
| 400410 | ST20 | 15q25.1 | Suppressor Of Tumorigenicity 20 | 6.1505 | 6.9169 |

**Table.S2. KEGG pathway cluster of DEGs**

| ID | KEGG Pathway | Count | PValue | Genes Symbol |
|---|---|---|---|---|
| hsa04640 | **Hematopoietic cell lineage** | 28 | 2.98E-05 | CD14, CD1A, CD1C, CD33, CD36, CD3D, CD3G, CD8B, CSF1R, CSF2RA, CSF3, CSF3R, FCER2, HLA-DRA, HLA-DRB1, HLA-DRB3, HLA-DRB4, HLA-DRB5, IL1R1, IL1R2, IL3RA, IL4, IL7R, ITGA2, ITGA2B, ITGA5, ITGAM, MME, THPO |
| hsa04612 | **Antigen processing and presentation** | 25 | 3.37E-04 | CD74, CD8B, CTSB, HLA-DMA, HLA-DPA1, HLA-DPB1, HLA-DQA1, HLA-DQA2, HLA-DQB1, HLA-DRA, HLA-DRB1, HLA-DRB3, HLA-DRB4, HLA-DRB5, HSPA1A, HSPA5, HSPA8, IFNA14, KIR2DL3, KIR2DL4, KIR2DS4, KLRC1, LGMN, PSME2, RFX5, TAP2 |
| hsa04514 | **Cell adhesion molecules (CAMs)** | 33 | 1.21E-03 | ALCAM, CD226, CD276, CD28, CD6, CD8B, CDH1, CDH15, CDH2, CDH4, CLDN14, HLA-DMA, HLA-DPA1, HLA-DPB1, HLA-DQA1, HLA-DQA2, HLA-DQB1, HLA-DRA, HLA-DRB1, HLA-DRB3, HLA-DRB4, HLA-DRB5, ICAM1, ITGAM, ITGAV, ITGB1, JAM3, L1CAM, NFASC, NLGN2, SDC3, SDC4, SELL, SPN |
| hsa04610 | **Complement and coagulation cascades** | 20 | 2.57E-03 | C1QA, C1QB, C1QC, C1R, C2, C3AR1, C5, C8B, CFD, CFH, CFI, F13A1, F2, F3, F7, PLAU, PLAUR, SERPINA5, TFPI, VWF |
| hsa05322 | **Systemic lupus erythematosus** | 24 | 9.72E-03 | C1QA, C1QB, C1QC, C1R, C2, C5, C8B, CD28, CTSG, FCGR2A, HIST1H2BC, HIST1H2BF, HIST1H2BO, HIST1H3G, HIST1H4C, HIST1H4I, HIST1H4J, HLA-DMA, HLA-DPA1, HLA-DPB1, HLA-DQA1, HLA-DQA2, HLA-DQB1, HLA-DRA, HLA-DRB1, HLA-DRB3, HLA-DRB4, HLA-DRB5 |
| hsa04060 | **Cytokine-cytokine receptor interaction** | 49 | 3.22E-02 | ACVR2B, CCL11, CCL13, CCL17, CCL18, CCL20, CCL21, CCL22, CCL23, CCL3L1, CCL5, CCL7, CCL8, CCR8, CSF1R, CSF2RA, CSF3, CSF3R, CXCL2, CXCL5, EDA2R, EGFR, FIGF, IFNA14, IFNAR1, IFNAR2, IL18, IL1R1, IL1R2, IL1RAP, IL22RA2, IL28RA, IL3RA, IL4, IL7R, IL8, IL8RB, INHBC, LIFR, LTB, NGFR, PDGFB, PPBP, PRLR, TNFRSF4, TNFSF13B, XCL1, XCL2, ZFP91 |
| hsa04620 | **Toll-like receptor signaling pathway** | 22 | 4.20E-02 | AKT2, CCL5, CD14, IFNA14, IFNAR1, IFNAR2, IKBKB, IL8, IRAK1, JUN, LBP, MAP2K6, MAP3K7IP2, MAP3K8, MAPK10, MAPK12, MAPK3, NFKBIA, PIK3CG, RAC1, SPP1, TICAM2 |
| hsa04062 | **Chemokine signaling pathway** | 36 | 4.69E-02 | ADRBK1, ADRBK2, AKT2, BCAR1, CCL11, CCL13, CCL17, CCL18, CCL20, CCL21, CCL22, CCL23, CCL3L1, CCL5, CCL7, CCL8, CCR8, CSK, CXCL2, CXCL5, GNGT2, IKBKB, IL8, IL8RB, MAPK3, NFKBIA, PAK1, PIK3CG, PPBP, PRKCB1, RAC1, RAP1B, STAT2, TIAM1, XCL1, XCL2 |

**Table.S3. Top Nodes in DEGs Signaling Nework**

| *Id* | *Label* | *Degree* | *Betweenness* |
|---|---|---|---|
| Q04206 | RELA | 65 | 2989.63 |
| P42224 | STAT1 | 50 | 218.86 |
| P40763 | STAT3 | 48 | 921.64 |
| Q9Y4K3 | TRAF6 | 44 | 1869.04 |
| P05412 | JUN | 43 | 1648.73 |
| P19838 | NFKB1 | 43 | 654.75 |
| O15111 | CHUK | 39 | 826.84 |
| Q12933 | TRAF2 | 38 | 724.53 |
| P00533 | EGFR | 36 | 1300.43 |
| P17676 | CEBPB | 34 | 449.98 |
| Q9Y6K9 | IKBKG | 33 | 520.26 |
| O14920 | IKBKB | 33 | 194.69 |
| Q06124 | PTPN11 | 32 | 546.11 |
| P29350 | PTPN6 | 31 | 947.64 |
| Q04864 | REL | 30 | 93.3 |
| P01106 | MYC | 29 | 1069.4 |
| P27986 | PIK3R1 | 29 | 0 |
| P23458 | JAK1 | 28 | 380.91 |
| O43318 | MAP3K7 | 28 | 0 |
| P19438 | TNFRSF1A | 25 | 556.83 |
| P42226 | STAT6 | 25 | 51.36 |
| O60674 | JAK2 | 24 | 329.28 |
| P02751 | FN1 | 23 | 1054.3 |
| Q13114 | TRAF3 | 23 | 178.67 |
| Q00653 | NFKB2 | 23 | 177.76 |
| O14543 | SOCS3 | 22 | 1005.94 |
| P28482 | MAPK1 | 22 | 572.08 |
| P45983 | MAPK8 | 22 | 323.94 |
| P45985 | MAP2K4 | 22 | 180.66 |
| Q99683 | MAP3K5 | 22 | 153.37 |

**Table.S4. Inhibitory receptor expression screen**

| *Entrez ID* | *Symbol* | *Other Name* | *Gene name* | *Cancer Exp1* | *Cancer Exp2* |
|---|---|---|---|---|---|
| 80381 | CD276 | B7H3 | CD276 molecule | 2.574 | 3.293 |
| 84868 | HAVCR2 | Tim3 | hepatitis A virus cellular receptor 2 | 2.199 | 2.475 |
| 5175 | PECAM1 | CD31 | platelet/endothelial cell adhesion molecule (CD31 antigen) | 1.557 | 1.427 |
| 4345 | CD200 | OX2 | CD200 molecule | 1.534 | 1.457 |
| 7301 | TYRO3 | | TYRO3 protein tyrosine kinase | 0.579 | 0.556 |
| 10288 | LILRB2 | ILT4 | leukocyte immunoglobulin-like receptor, subfamily B (with TM and ITIM domains), member 2 | 0.522 | 0.374 |
| 50856 | CLEC4A | DCIR | C-type lectin domain family 4, member A | 0.485 | 0.458 |
| 2212 | FCGR2A | FcγRIIA | Fc fragment of IgG, low affinity IIa, receptor (CD32) | 0.259 | 0.188 |
| 10462 | CLEC10A | CD301 | C-type lectin domain family 10, member A | 0.158 | 0.123 |

**Table.S5. qRT Primers**

| *Gene* | *Forward Primer* | *Reverse Primer* |
|---|---|---|
| **Actin** | CATGTACGTTGCTATCCAGGC | CTCCTTAATGTCACGCACGAT |
| **GAPDH** | CCATGTTCGTCATGGGTGTGAACCA | GCCAGTAGAGGCAGGGATGATGTTC |
| **HLA-DMA** | TGATCCAGCAAATAGGGCCA | CTCTGGACACCGGGATTTTC |
| **HLA-DMB** | AAAGACACCCTGATGCAGCG | TGTGGCACAATTCTGAAGCC |
| **HLA-DOA** | TGGCCCAGACCAGCTTCTAT | GGAACTTGCGGAACAAATGG |
| **HLA-DPB** | GGGACACAGCGCTTCCTGGAG | CAAGCAGGTTGTGGTGCTGCA |
| **HLA-DQA** | CTCTGACCACCGTGATGAGC | CTCTCCAGGTCCACGTAGAA |
| **HLA-DQB** | CGAGTACTGGAACAGCCAGAAGG | GGAGTCATTTCCAGCATCACCAGG |
| **HLA-DRB** | CACCAGACCACGTTTCTTGGAGT | CACGTTCTCCTCCTGGTTATGGA |
| **CD74** | TGGAGAACCTGCGCATGAAG | CTTGGTGGCATTCTGCATGG |
| **CIITA** | CCTGCTGTTCGGGACCTAAA | GGATCCGCACCAGTTTGG |
| **IL1b** | AGCTACGAATCTCCGACCAC | CGTTATCCCATGTGTCGAAGAA |
| **IL1R1** | ATGAAATTGATGTTCGTCCCTGT | ACCACGCAATAGTAATGTCCTG |
| **IL1R2** | ATGTTGCGCTTGTACGTGTTG | CCCGCTTGTAATGCCTCCC |
| **IL1RAP** | GGGGACTAGACACCATGAGG | ACCAGATCAGAGTAAGGCCAG |
| **IL1RN** | CATTGAGCCTCATGCTCTGTT | CACTGTCTGAGCGGATGAA |
| **IL6** | CCAGGAGCCCAGCTATGAAC | GATGCCGTCGAGGATGTACC |
| **IL10** | TCAAGGCGCATGTGAACTCC | GATGTCAAACTCACTCATGGCT |
| **IFNG** | TCGGTAACTGACTTGAATGTCCA | TCGCTTCCCTGTTTTAGCTGC |
| **TNF** | CCTCTCTCTAATCAGCCCTCTG | GAGGACCTGGGAGTAGATGAG |
| **NFKB1** | AACAGAGAGGATTTCGTTTCCG | TTTGACCTGAGGGTAAGACTTCT |
| **RelA** | ATGTGGAGATCATTGAGCAGC | CCTGGTCCTGTGTAGCCATT |
| **IRF8** | AGTAGCATGTATCCAGGACTGAT | CACAGCGTAACCTCGTCTTC |
| **MafB** | GACGCAGCTCATTCAGCAG | CCGGAGTTGGCGAGTTTCT |
| **STAT2** | GAGCCAGCAACATGAGATTGA | GCCTGGATCTTATATCGGAAGCA |
| **TAP1** | TCTCCTCTCTTGGGGAGATG | GAGACATGATGTTACCTGTCTG |
| **TRAF2** | GCTCATGCTGACCGAATGTC | GCCGTCACAAGTTAAGGGGAA |
| **TRAF3** | GCGTGTCAAGAGAGCATCGTT | GCAGATGTCCCAGCATTAACT |
| **BIC** | TGGCTCTCCCACCCAATGG | AGAATTTAAACCACAGATTTCC |
